# Objective quantification of nerves in immunohistochemistry specimens of thyroid cancer utilising deep learning

**DOI:** 10.1371/journal.pcbi.1009912

**Published:** 2022-02-28

**Authors:** Indriani P. Astono, James S. Welsh, Christopher W. Rowe, Phillip Jobling

**Affiliations:** 1 School of Engineering, The University of Newcastle, Newcastle, Australia; 2 School of Medicine and Public Health, The University of Newcastle, Newcastle, Australia; 3 School of Biomedical Sciences and Pharmacy, The University of Newcastle, Newcastle, Australia; Harvard University, UNITED STATES

## Abstract

Accurate quantification of nerves in cancer specimens is important to understand cancer behaviour. Typically, nerves are manually detected and counted in digitised images of thin tissue sections from excised tumours using immunohistochemistry. However the images are of a large size with nerves having substantial variation in morphology that renders accurate and objective quantification difficult using existing manual and automated counting techniques. Manual counting is precise, but time-consuming, susceptible to inconsistency and has a high rate of false negatives. Existing automated techniques using digitised tissue sections and colour filters are sensitive, however, have a high rate of false positives. In this paper we develop a new automated nerve detection approach, based on a deep learning model with an augmented classification structure. This approach involves pre-processing to extract the image patches for the deep learning model, followed by pixel-level nerve detection utilising the proposed deep learning model. Outcomes assessed were a) sensitivity of the model in detecting manually identified nerves (expert annotations), and b) the precision of additional model-detected nerves. The proposed deep learning model based approach results in a sensitivity of 89% and a precision of 75%. The code and pre-trained model are publicly available at https://github.com/IA92/Automated_Nerves_Quantification.

## Author summary

The study of nerves as a prognostic marker for cancer is becoming increasingly important. However, accurate quantification of nerves in cancer specimens is difficult to achieve due to limitations in the existing manual and automated quantification methods. Manual quantification is time-consuming and subject to bias, whilst automated quantification, in general, has a high rate of false detections that makes it somewhat unreliable. In this paper, we propose an automated nerve quantification approach based on a novel deep learning model structure for objective nerve quantification in immunohistochemistry specimens of thyroid cancer. We evaluate the performance of the proposed approach by comparing it with existing manual and automated quantification methods. We show that our proposed approach is superior to the existing manual and automated quantification methods. The proposed approach is shown to have a high precision as well as being able to detect a significant number of nerves not detected by the experts in manual counting.

This is a *PLOS Computational Biology* Methods paper.

## 1 Introduction

A growing number of studies have shown that nerves are involved in the initiation and progression of numerous cancers [1]. For example, in prostate, breast, pancreatic and bowel cancers, the presence of nerves has been associated with increased tumour aggressiveness and a higher potential for metastatic spread [2–4]. With respect to thyroid cancer, it has been shown that nerve density is higher in papillary thyroid cancer as compared to follicular thyroid cancers and benign thyroid tissues [5].

The detection of nerves in tissue sections presents challenges. Large nerve trunks that distribute the axons of central neurons to their peripheral targets are relatively easy to identify. However, the terminal fields of neurons consist of individual fine axons with diameters between 500 nm and 1 *μ*m. As such, experts need to use specific neuronal markers and examine specimens at high magnification to identify them. This narrows the field of view and makes manual quantification challenging.

A number of manual counting strategies exist, in which small regions of interest are first identified, followed by counting of the stained nerves (as determined by immunohistochemical labelling of specific neuronal markers) by a trained expert in these regions [2, 5–7] or in a random selection of these regions [3, 4]. Automatic methods, e.g. computerised nerve planimetry, involve an expert manually selecting a few regions of interest centred around the target observation to define a colour filter range in each image. The image is then converted to a binary format using the colour filter, where the planimetry of the total nerve surface area will be determined [6, 7].

Manual counting has high precision. However, it is labour-intensive and usually has a low sensitivity [8]. It is also susceptible to bias and inconsistency due to intra- and inter-expert variability, as fatigue and other external factors may affect an expert’s judgement [8]. On the other hand, computerised nerve planimetry is very fast and has a high sensitivity, but typically has a very low precision. It should be noted that some studies, that use computerised nerve planimetry, use manual region definition prior to planimetry analysis [6, 7].

**Fig 1:**
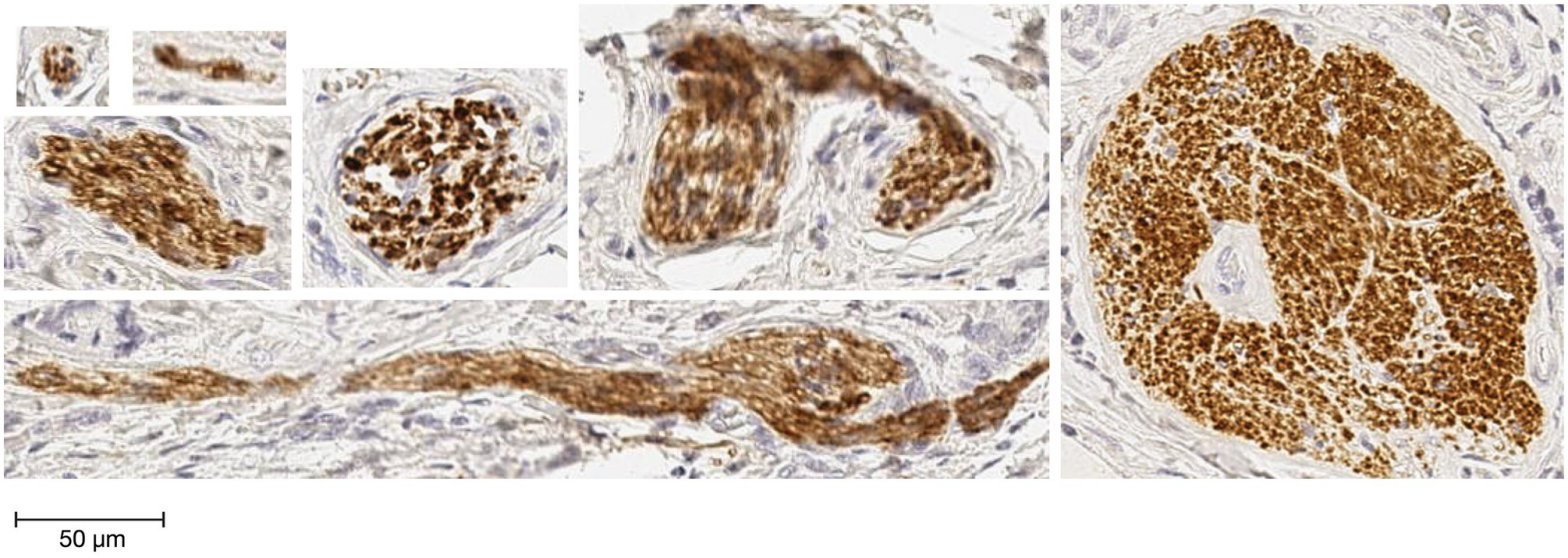
Examples of variation in the size of nerves.

**Fig 2:**
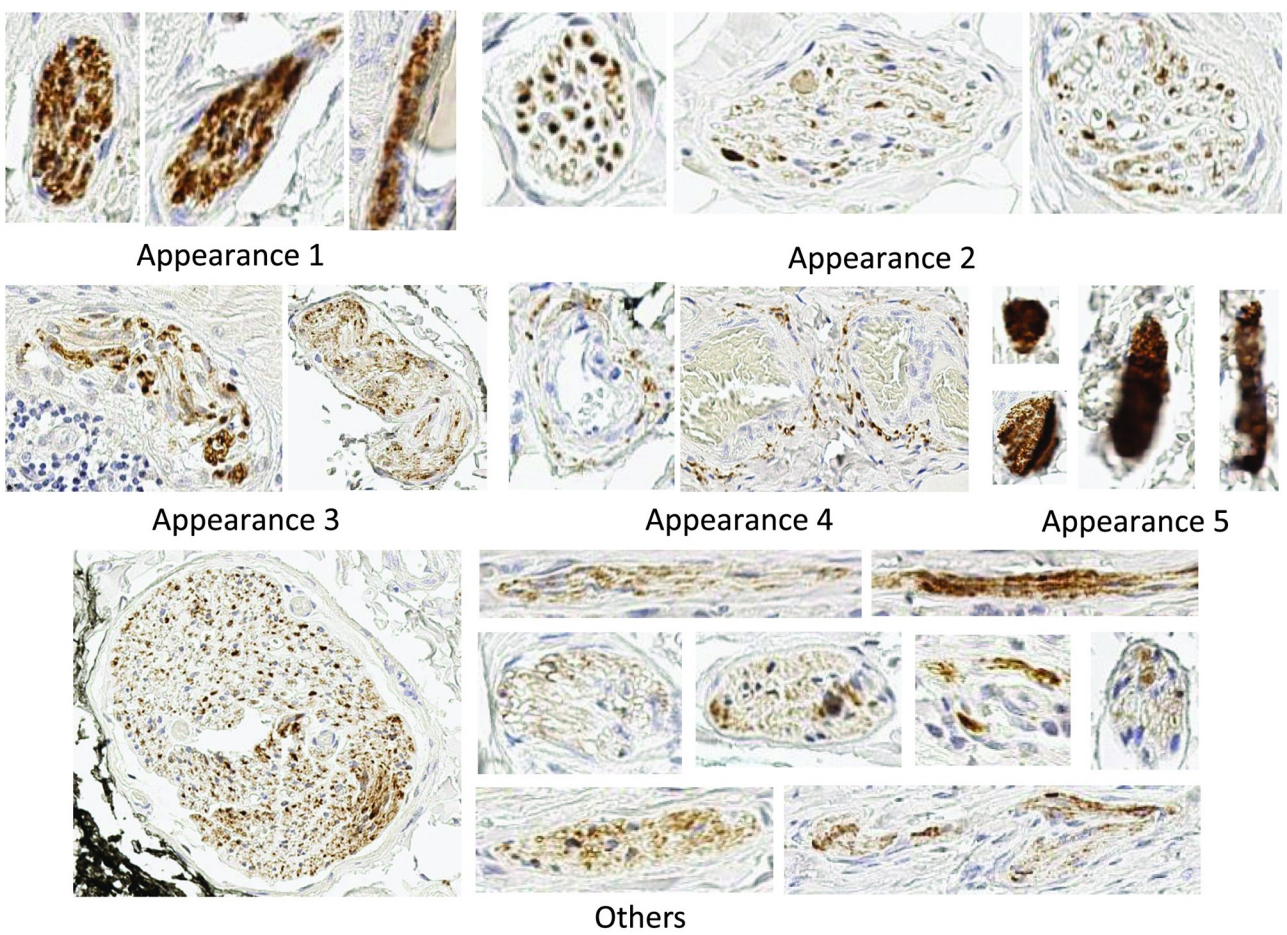
Examples of variation in the appearance of nerves.

Nerves vary largely in size and appearance. Fig 1 contains several examples that show variation in the size of nerves resulting from different ways in which a nerve can be observed in an immunohistochemical sample, while Fig 2 contains a number of different nerve appearances. An automatic detection algorithm must be able to competently identify, considering all of these different sizes and appearances, a nerve.

**Fig 3:**
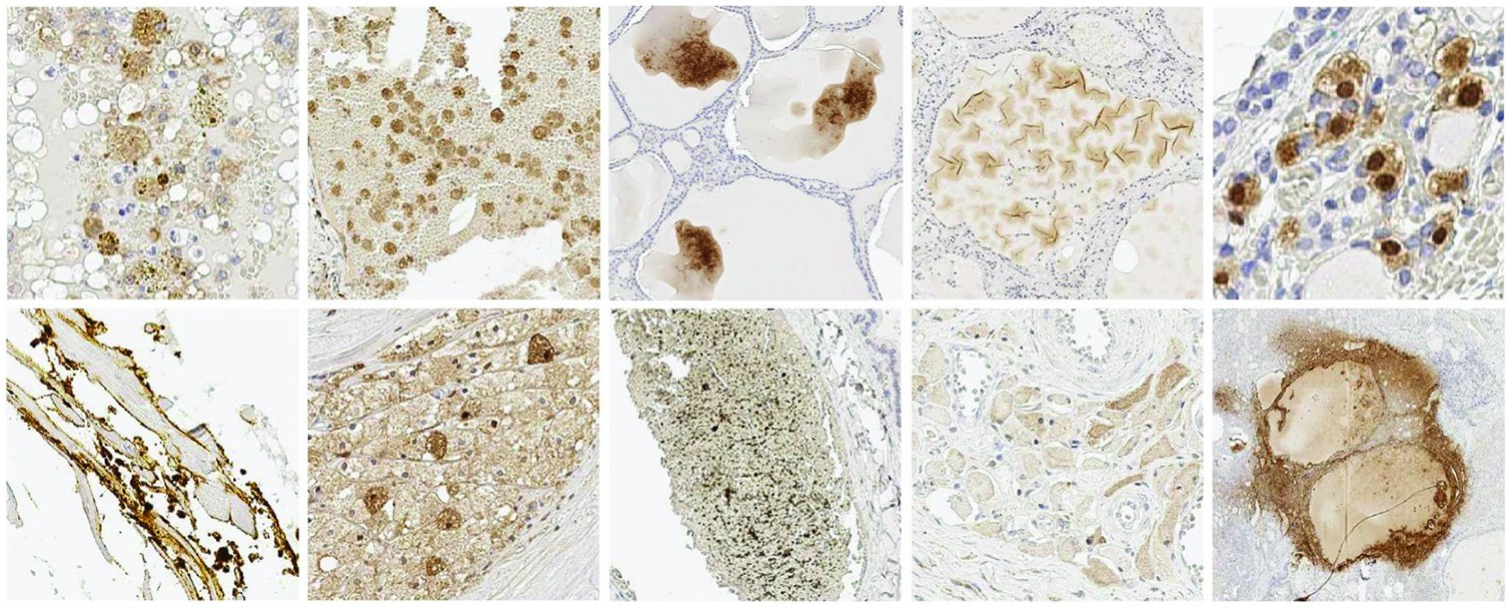
Examples of non-specific staining.

As can be seen from both figures, the main identifier of the nerves is the immunohistochemical labelling that gives the colour contrast to the nerves, brown in this case. Unfortunately, the colour cannot be solely used as the nerve detection criteria. A key problem is that even the most specific immunohistochemical labelling of nerve proteins inevitably has some degree of non-specific labelling of background tissue that contributes to false positive nerve detection. There are many different types of non-specific staining, a selection of which are shown in Fig 3. As can be seen from Figs 1, 2 and 3, the differences between non-specific staining and the nerves are not obvious in terms of colour, size and appearance. This non-specific staining results in the nerves being difficult for the experts to distinguish and also leads to an overestimation of the number of nerves by the existing automatic methods.

The variation of nerves in terms of size and appearance also results in no formal definition of a standardised nerve detection criteria. As a result, it is difficult to compare detection and quantification methods across studies. It can be seen in Fig 1 that the size of a nerve can vary from 25*μm*^2^ to 22, 500*μm*^2^. While, in Fig 2, it can be seen that nerves have many different appearances, e.g. a nerve can appear as a single formation (appearance 1), separated small formations (appearance 2), separated formations (appearance 3), separated clusters (appearance 4), smudges (appearance 5) or other appearances (appearance 6). Although a nerve trunk can be easily detected due to its characteristic morphology and location features, a cluster of axons is more difficult to objectively quantify. A non-standardised detection criteria makes the development of an objective approach difficult.

A significant challenge is also presented by the very large image size, i.e. 40, 000 × 40, 000 pixels or larger, of a digital whole-slide section. The large image size causes fatigue in experts and a large computational time in computerised methods.

A convolutional neural network (CNN) is a type of deep learning model that has the ability to recognise patterns and learn from a set of data samples to make sensible predictions for new data samples. Deep learning is an automated method that has a feature-learning capability that allows the system to extract features directly from an input image. By simply providing the desired output, a deep learning model can be used to automatically learn from a set of data samples directly. However, data preparation and training processes should be carefully designed to ensure that the learning is effective. It is also a challenge to develop a deep learning model with incomplete expert annotations and coarsely annotated data, as is the case in this paper, where the data used is from a recent study [5] where many smaller nerves are not detected or annotated.

In this paper, we propose a nerve detection approach that uses a CNN to improve nerve quantification for thyroid cancer biomarker studies. The main contribution is in the development of an objective nerve detection methodology based on a segmentation network incorporating a novel augmented classification structure. We evaluate our proposed CNN based approach in performing the nerve detection and quantification task on data made available from the study in [5].

In Section 2, we provide some background material for the proposed nerve detection approach. In Section 3, we describe the tissue preparation and digitisation process, nerve detection criteria, segmentation label extraction process, performance evaluation metrics and details of our proposed approach, including the proposed architecture, and training process. In Section 4, we describe the dataset and compare the performance of our proposed model to manual counting and a colour filter only based approach for the nerve detection and quantification task. In Section 5, we discuss the results of our study and challenges in the development of the proposed approach. Finally, we present conclusions in Section 6.

## 2 Related work

Many CNN based approaches have been applied to object detection tasks, for example, object detection in photographs [9–11], organ detection in medical images [12, 13] or mitosis, cytoplasm and nuclei detection in stained whole slide images (WSIs) [14–16]. However, to our best knowledge, no CNN based approach has been applied specifically to the nerve detection and quantification problem. In this section we will present the rationale for our approach based on some object detection problems that are similar to this nerve detection problem.

### 2.1 Colour thresholding

In image processing it is typical to convert an image from the red, green and blue (RGB) colour space to the hue, saturation and value (HSV) colour space for the purpose of colour image segmentation and/or thresholding [17]. This is because the HSV colour space organises colour in a similar way to the perception of the human eye [17], in that luma/intensity information are separated from chroma/colour information in the HSV colour space [18]. This makes colour range definition in the HSV colour space more straight forward in comparison to the RGB colour space.

To perform colour thresholding (i.e. filtering) on a WSI in a typical image processing program, e.g. ImageJ [19], an expert takes a sample of the target instances to initialise the colour filter range in the HSV colour space. Then, the expert will adjust the threshold limit manually until the desired segmentation output is obtained.

### 2.2 Object detection and localisation in a WSI

In computer vision, an object quantification task is usually formulated as an object detection or localisation task [20, 21]. Some of the most successful approaches in object detection and localisation evolved from relying on either a multi-scale sliding-window (i.e. exhaustive search) [22–24], a selective-search [9, 10, 25, 26] or deep learning models [9–11, 22–26].

Deep learning models such as U-Net and FCN have been shown to be successful in various WSI segmentation applications [27], with U-Net proven to be superior [28]. U-Net has also been shown to outperform human experts for lymphocyte detection in immunohistochemically stained tissue sections of breast, colon and prostate cancer [28]. The superiority of U-Net performance with respect to the FCN has also been demonstrated on the application of renal tissue segmentation [29].

### 2.3 Supervised learning

To develop a supervised learning model for segmentation, including a CNN, a complete pixel-wise annotated dataset is required as the ideal supervision information [30]. However, as a complete pixel-wise annotated dataset is often unavailable in real-world applications, a basic assumption, e.g. a cluster assumption or a manifold assumption, can be adopted to annotate the non-annotated data for training [30].

It is also crucial to ensure that the data for each class is balanced, i.e. intra- and inter-class data [31] [32]. In the case where the data is highly skewed with respect to the number of annotations in each class, the training data samples should be carefully selected to ensure that the representations of each class are balanced [32]. Data balancing can be achieved by controlling the proportion of the data samples of each class to be equal [32].

## 3 Materials and methods

### 3.1 Ethics statement

This study was approved by the Hunter New England Human Research Ethics Committee, who granted a waiver of consent for access to archival pathology material and approved the experimental protocol (2019/ETH13695). All study methodologies were carried out in accordance with relevant guidelines and regulations.

### 3.2 Tissue preparation and digitisation

The images used in this study are from a dataset of histological specimens of benign and malignant thyroid tissue that has previously been described [5]. This dataset [33] was chosen, firstly, because of the high specificity of the immunohistochemistry for nerve tissue with minimal background staining, and secondly, because a digital library of annotated nerves was readily available. Briefly, 112 whole slide sections of 4 *μ*m thickness, from formalin-fixed paraffin-embedded blocks of benign and malignant thyroid tissue, were labelled with immunohistochemistry for the pan-neuronal marker protein gene-product 9.5 (PGP9.5) using the Ventana Discovery automated slide stainer (Roche Medical Systems, Tuscon, Az), then counterstained with haematoxylin. The primary antibody was the anti-rabbit polyclonal PGP9.5 antibody (catalogue number #Ab15503, Abcam, Cambridge, United Kingdom) at 1:600 dilution. Slides were then digitised at 20 × magnification using the Aperio AT2 scanner (Leica Biosystems, Victoria, Australia). These images were analysed using the QuPath quantitative pathology program [34]. Slides were initially viewed at 4x magnification as specified by the program and screened for the presence of focal DAB staining suggestive of nerves. All focal DAB staining was then examined at 20x magnification and ascribed the appropriate annotation.

### 3.3 Criteria for nerve detection

As discussed in Section 1, nerve detection is considered to be a difficult task as nerves vary in size, appearance and immunoreactivity to the PGP9.5 staining colour range. However, it is critical to define comprehensive criteria to reduce bias in the study. Thus, we develop the criteria based on four parameters: size, morphology, anatomical location and immunoreactivity to PGP9.5. The definitions of the criteria are given in Table 1.

**Table 1:** Criteria for nerve detection.

Criterion	Definition	Justification
Anatomical location	Plausible anatomical location for neural tissue (e.g. vasa nevorum, interstitial spaces)	Structures outside of a plausible anatomical location are excluded.
Size	Greater than 25*μ*m^2^ (∼100 pixels)	Corresponds to a minimum size of 3 axons in cross-section. Smaller structures are difficult to confidently distinguish from non-specific staining
Morphology	Typical neural structures	Axon: linear structure, discrete edge
		Nerve: cluster of axons surrounded by perineurium
Immunoreactivity	Focal and discrete PGP9.5 staining	Diffuse and non-specific uptake of Dab are excluded.

As can be seen from Table 1, a nerve should be in a plausible anatomical location and have a minimum size of 25 *μ*m^2^ (∼100 pixels). In terms of appearance and colour, a nerve should show immunoreactivity to PGP9.5 staining, i.e. be brown in colour, and show typical neural structure, e.g. edges, clearly.

The minimum size of the manually annotated nerve in the dataset from [5] is approximately 100 *μ*m^2^ (400 pixels), which is about four times the minimum nerve size that we would like to detect. Hence, we use a 20 × 20 pixel (400 pixels) morphological closing operation [35] to combine predicted positive instances located close to each other and consider it as a single predicted positive instance. Predicted positives instances that are far from each other (e.g. axons of a nerve cluster) and that cannot be morphologically closed, will be counted as discrete predicted positive instances.

### 3.4 Segmentation label extraction

Here we are faced with the problem of incomplete coarsely annotated data of only true positives. Thus, we use assumptions, based on colour and location, to determine the pixel-wise segmentation labels of the coarsely annotated data to maximise the use of non-annotated data for learning, as discussed in Section 2.3. If a pixel is brown in colour and intersects with the annotations, it is labelled positive, otherwise it is labelled negative.

A significant challenge is in determining the colour filter range for the pixel-wise segmentation labels that results in a minimum number of label artifacts. It is difficult to determine a colour filter range that can detect the immunoreactivity to PGP9.5 staining with high sensitivity and high precision (i.e. high true positives and low false positives), as a colour filter range with high sensitivity usually results in low precision. Moreover, all the true positives and false positives cannot be determined from incomplete annotations. Thus, the range is defined empirically by observing the number of true positives (i.e. detected annotations) and estimated false positives (i.e. label artifacts from detected non-annotated brown stains). A colour filter predicted positive instance is considered to be a true positive if the predicted positive instance intersects with any of the annotations. Here we use a normalised colour filter range of (0.04, 0.2, 0.4) to (0.2, 1, 1) in HSV space that results in approximately 98% sensitivity, with respect to the expert annotated data, to minimise the label artifacts.

Another challenge is to extract true negative training data samples. With incomplete coarsely annotated data of only true positives, the negative data samples cannot be extracted reliably. There are many nerves that were falsely annotated negative by exclusion in the expert-annotated data. Therefore the colour filter predicted positive instances located outside the annotations cannot be used directly as negative training data samples. To solve this problem, we define an area within each training WSI where negative training data samples are to be extracted. The regions are chosen such that the number of unspecified brown stains is maximised and the number of false negatives is minimised.

### 3.5 Performance evaluation

The main objective of this paper is to provide an automatic approach for the quantification of nerves in a thyroid tissue WSI. From this perspective, it is important to evaluate the proposed approach at a WSI level instead of at a patch level. As there is no precise pixel-wise label available, the performance will be evaluated by a hit or miss method. A hit indicates a true positive, while a miss indicates either a false negative or false positive. A hit is determined when a predicted positive instance intersects with an expert annotation, i.e. true positive from manual annotations, *TP*_*m*_. When multiple predicted positive instances intersect with an expert annotation, it will be scored as one hit. An expert annotation that has no intersection with any predicted positive instance will be scored as a miss, i.e. false negative, *FN*. If a predicted positive instance does not intersect any of the expert annotations, a hit is then determined by experts, who evaluate whether the predicted positive contains any nerve, i.e. additional detection true positive, *TP*_*a*_. If the predicted positive instance contains no nerve, as determined by the experts, it will be scored as a miss, i.e. false positive, *FP*. The evaluation (scoring) is performed blindly by three experts, i.e. E1, E2 and E3, and the final number of true positives will be determined from the average across the experts. The inter-expert agreement is shown in Table 2. Overall, the percentage of the scoring agreements between the experts (E1 and E2, E1 and E3, and E2 and E3) has an average of 78.3%, which is broadly consistent with the literature [36, 37].

**Table 2:** Inter-expert scoring agreement.

Experts	Average Agreement (%)
**E1,E2**	74.9
**E1,E3**	79.3
**E2,E3**	80.6
**Average**	**78.3**

Sensitivity, also referred to as true positive rate (*TPR*), is used to evaluate the number of annotated nerves detected. Sensitivity [38] is given by,

$$\begin{array}{c} TPR=\frac{TP_{m}}{\left(TP_{m}+FN\right)}, \end{array}$$ (1)

where *TP*_*m*_ indicates the number of true positives detected from the manual annotations and *FN* indicates the number of false negatives.

Precision, also referred to as positive predictive value (*PPV*), is used to evaluate the ability of an approach to detect nerves. Precision [38] is given by,

$$\begin{array}{c} PPV=\frac{TP}{\left(TP+FP\right)}, \end{array}$$ (2)

where *TP* indicates the total number of true positives,

$$\begin{array}{c} TP=TP_{m}+TP_{a}, \end{array}$$ (3)

*TP*_*a*_ indicates the number of true positives in the additional detections, i.e. nerves missed by the experts in the manual annotations, and *FP* indicates the number of false positives.

### 3.6 Nerve detection approach

**Fig 4:**
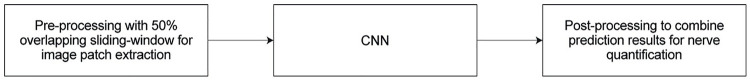
End-to-end process flowchart.

The nerve detection approach consists of three main stages, preprocessing, model prediction and post-processing. The end-to-end process is shown in Fig 4.

The approach starts with a sliding-window having a 50% overlap that is used to extract 160 × 160 pixel image patches from the entire WSI. Then, a CNN is used to perform pixel-wise segmentation of the nerve in each image patch before the predictions are combined for nerve quantification.

The output of the network is the same size as the input and is a binary array of pixel-wise predictions. However, the output may contain predicted positive instances that are too small, or a cluster of predicted positive instances that are separated from each other. Hence, a post-processing process is required to omit the small predicted positive instances below the minimum size threshold, or to combine the predicted positive instances into a larger instance for better nerve quantification.

**Fig 5:**
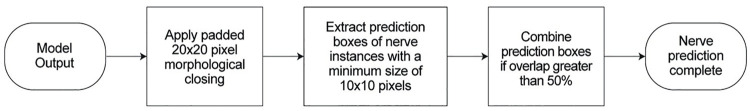
Post-processing flowchart.

The post-processing begins with a 20 × 20 pixel morphological closing [35] that combines the predicted positive instances that are located closer than 20 pixels in both horizontal and vertical axes. For each of the predicted positive instances the minimum x and y coordinate is determined and the maximum width and height of the instance is calculated. The box is then generated around the predicted positive instance where one corner is located at the minimum coordinate and the box is given the width and height of the calculated maximum values. Then, the predicted positive instances with prediction boxes that are larger than 100 pixel are extracted, while smaller instances are omitted. Finally, the prediction boxes that overlap by at least 50% are combined and counted as one, while two prediction boxes that overlap less than 50% are counted as two individual nerves. The post-processing flowchart is shown in Fig 5.

### 3.7 Proposed architecture

In this section, we propose the novel addition of a classification structure to the U-Net architecture [39] as the nerve detection task involves an image classification process. Although a multi-stage classification and segmentation approach can be used, the training can be computationally expensive, as separate training processes are required. Thus, we propose to augment the U-Net architecture [39] with an image classification structure that can be trained in an end-to-end manner to improve the segmentation results.

**Fig 6:**
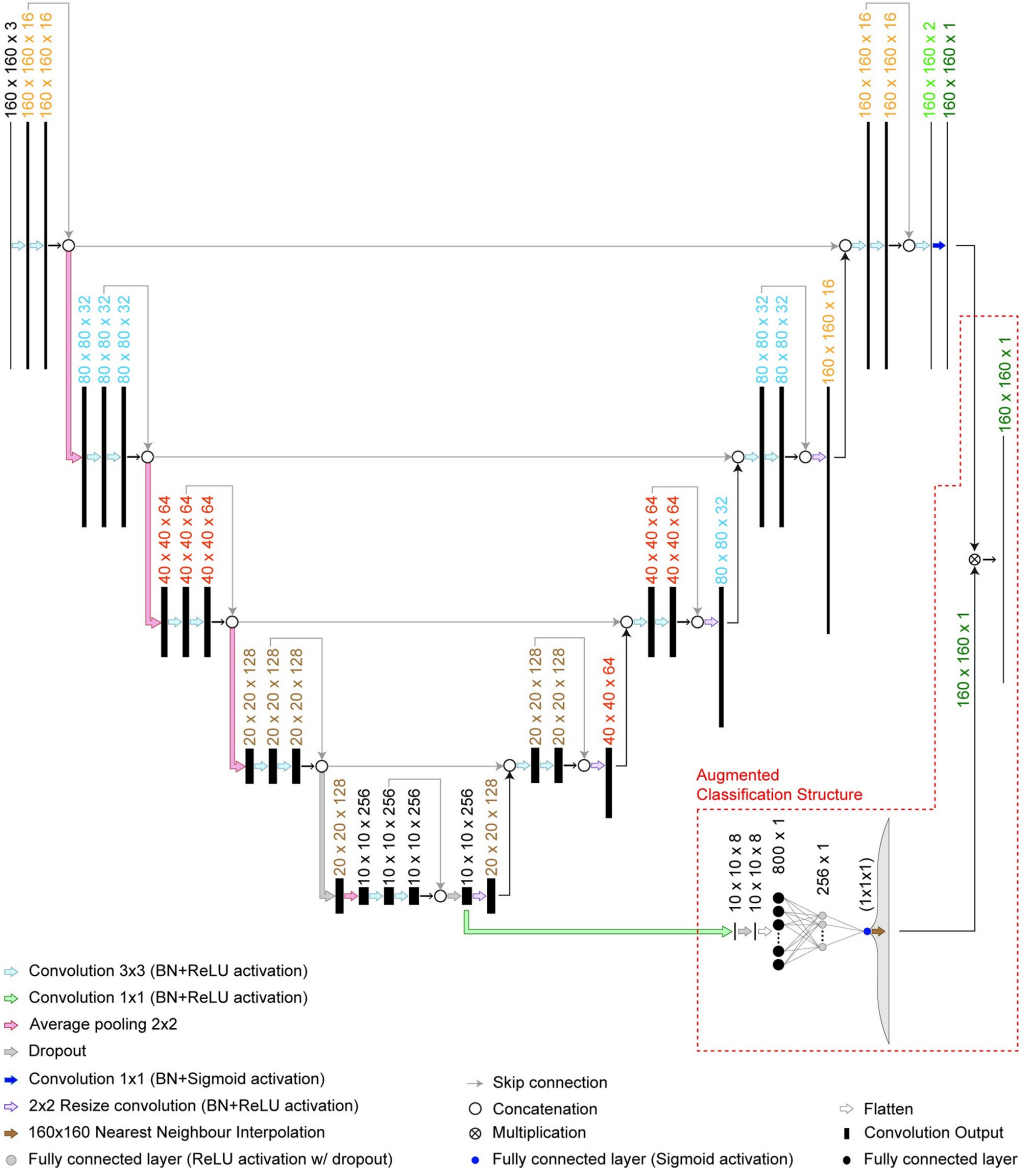
Proposed network architecture with augmented classification structure.

The U-Net architecture [39] uses an encoder-decoder structure to perform pixel-wise classifications. The encoder is used for automatic feature extraction of the input image, while the decoder is used to map the extracted high-level features back to the original input resolution and use them to perform pixel-wise classification for the segmentation output. The augmented classification structure is designed to use the extracted high-level features for image classification and then have the classification output assist the final segmentation results. The aim of the augmented classification structure is to reduce the false positive predictions of the segmentation network. The proposed network architecture with the augmented classification structure is shown in Fig 6.

As shown in Fig 6, the U-Net based network [39] begins with a 160 × 160 × 3 input layer and ends with a 160 × 160 × 1 sigmoid activated output layer. It consists of convolution blocks containing 3 × 3 convolution layers with ReLU activation. A 2 × 2 average pooling layer is used for the downsampling in the encoder and a 2 × 2 nearest neighbour interpolation followed by 2 × 2 convolution layer with ReLU activation is used for the upsampling in the decoder. Skip connections with concatenation are used to pass feature maps within each convolution block as well as from the encoder to the decoder. Dropout layers, with dropout rate of 0.5, are applied in the lowest resolution convolution block for regularisation. Batch normalisation (BN) layers are applied before every activation layer to normalise the input and speed up the training process.

**Fig 7:**
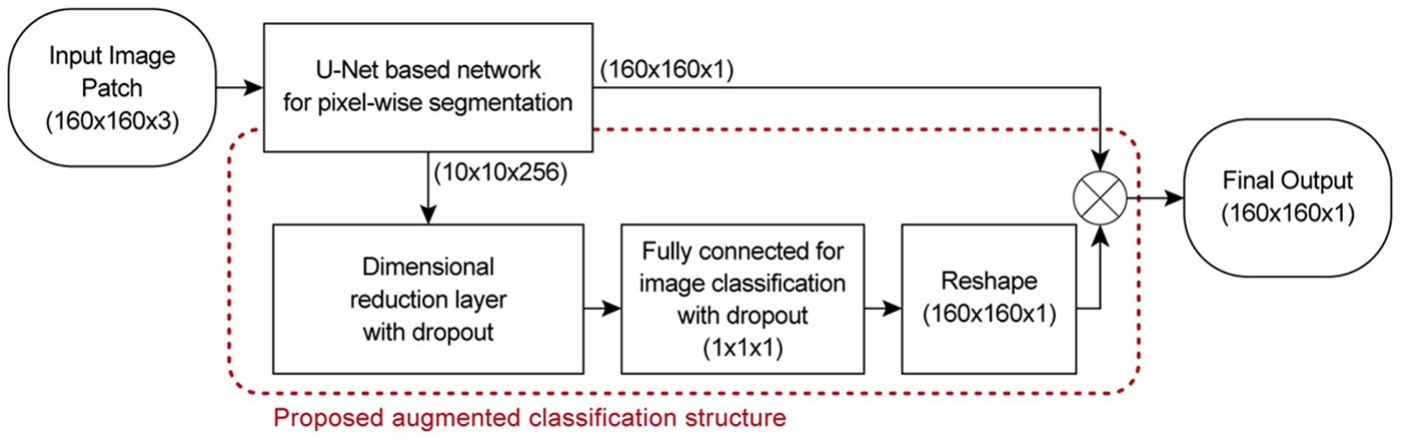
Block diagram of the proposed network with augmented classification structure.

The augmented classification structure consists of a 1 × 1 convolution layer for a feature layer dimensional reduction, a 2-layer fully connected network for classification and a reshaping layer for the classification output shape adjustment. The reshaping layer takes the 1 × 1 classification output (with probability range of 0 to 1) and reshapes it to the input image size of 160 × 160 by duplication. A dropout layer, with a dropout rate of 0.5, is applied directly after the 1 × 1 convolution layer and before the last fully connected layer for regularisation. The reshaped output is then multiplied by the output of the segmentation network to obtain the final model output which has a threshold of 0.5. This results in a segmentation network that only produces a positive segmentation output when the results of the classification and segmentation output are higher than 0.5. The block diagram of the proposed network with the augmented classification structure is shown in Fig 7.

### 3.8 Training

**Fig 8:**
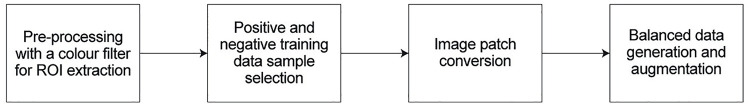
CNN training process.

The training consists of four main stages, pre-processing with a colour filter for ROI extraction, training data sample selection, image patch conversion and training data generation. The training process is shown in Fig 8.

**Fig 9:**
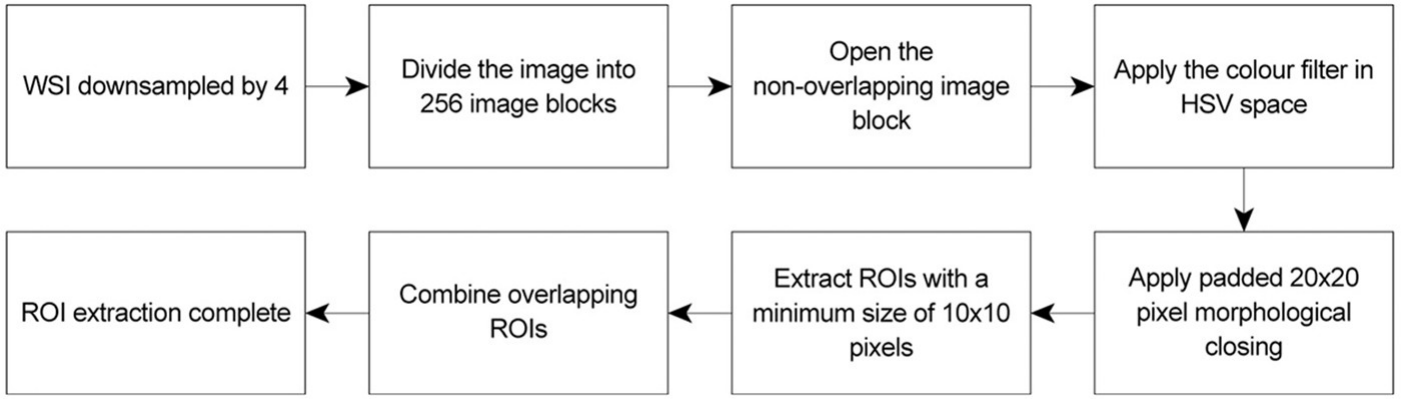
ROI extraction flowchart.

Pre-processing includes filtering based on colour for the extraction of ROIs and network input preparation. For the extraction of the ROIs, we downsampled the WSI by 4 to reduce the size of the image to approximately 10, 000 × 10, 000 pixels. The WSI is then divided into 256 non-overlapping image blocks, where each image block consists of approximately 625 × 625 pixels. We then apply the colour filter described in Section 3.4. Note that the binary output of the colour filter is quite noisy, as it catches many small artifacts of brown stains that do not belong to a nerve. A 20 × 20 pixel morphological closing is performed to combine brown stains that potentially belong to a nerve, i.e. separated axons in a nerve fibre or nerve trunks, and extract the resulting structures that exceed 100 pixels as ROIs. Finally, we combine ROIs that overlap each other to form a larger ROI. The ROI extraction flowchart is shown in Fig 9.

**Fig 10:**
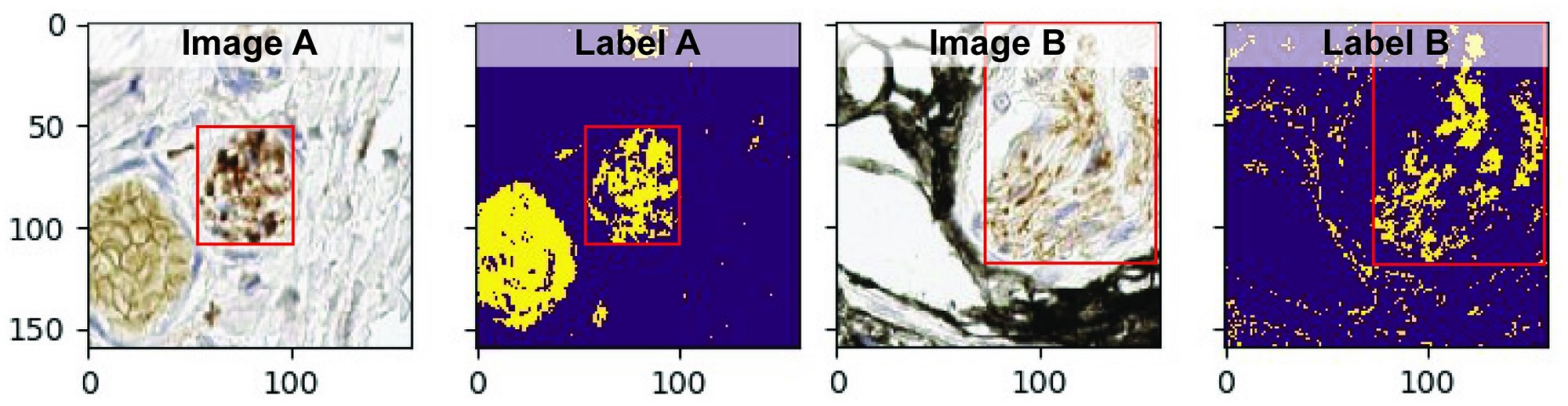
Excluded positive training data samples due to label artifacts from the colour filter. The red box surrounds the actual nerve.

After the ROIs have been extracted from the WSI, they are categorised into positive and negative training data samples. We use the same method to determine a true positive as in Section 3.5, where any ROI that intersects with any of the expert annotations will be considered as positive training data samples, while any ROI that intersects with the predefined areas for the extraction of the negative training samples will be considered as negative training data samples. To ensure accurate representation of the target class, i.e. nerves, the ∼2500 positive training data samples were manually examined and approximately 500 positive training data samples that contain a significant number of label artifacts were excluded. Examples of excluded positive training data samples due to label artifacts are shown in Images A and B of Fig 10, while the pixel-wise segmentation labels of the corresponding image patches are shown in Labels A and B of Fig 10. As can be seen in these examples, a significant number of label artifacts, i.e. positive pixel-wise labels located outside the red box, are apparent in the form of a blob, Label A, and scattered structure, Label B.

**Fig 11:**
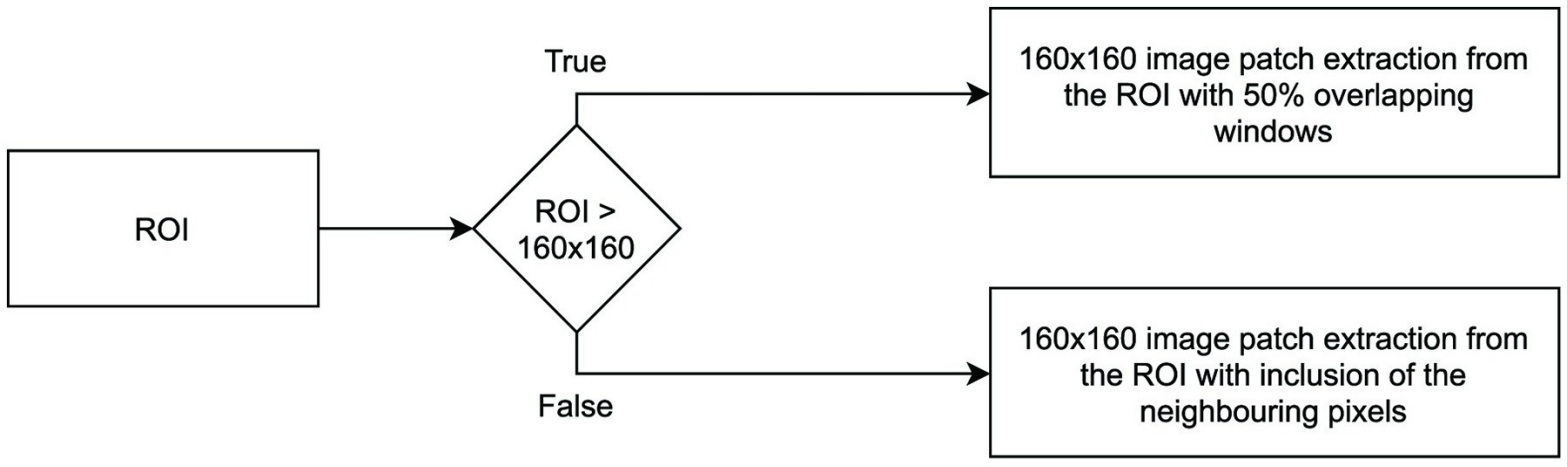
Image patches conversion flowchart.

The CNN accepts a fixed 160 × 160 pixel, 3 channel (RGB) image. Due to the variation in the size of the nerves, we convert each of the arbitrary size ROIs to 160 × 160 pixel image patches. To accomplish this, we use the process shown in Fig 11. Here, we divide the ROI into 50% overlapping, 160 × 160 pixel image patches without the use of any reshaping function. Basically, we check if the size of the ROI is greater than 160 × 160 pixels, and if true, we divide the ROI height and width with the image patch size and obtain the number of image patches that will be extracted from the ROI. The number of image patches extracted from each ROI can be calculated as follows,

$$\begin{array}{c} N=\lceil \frac{ROI}{P}\rceil \times 2-1, \end{array}$$ (4)

where *N* denotes the number of 50% overlapping patches that are required to cover the size of either the ROI height or width, and *P* denotes either the size of the patch height or width.

As discussed in Section 2.3, to ensure the training is effective, a balanced dataset is required. Here a training generator is used to perform data augmentation (e.g. flip, rotate and shifting) and generates balanced training data samples, where an equal number of positive and negative data samples are randomly chosen at every iteration.

**Fig 12:**
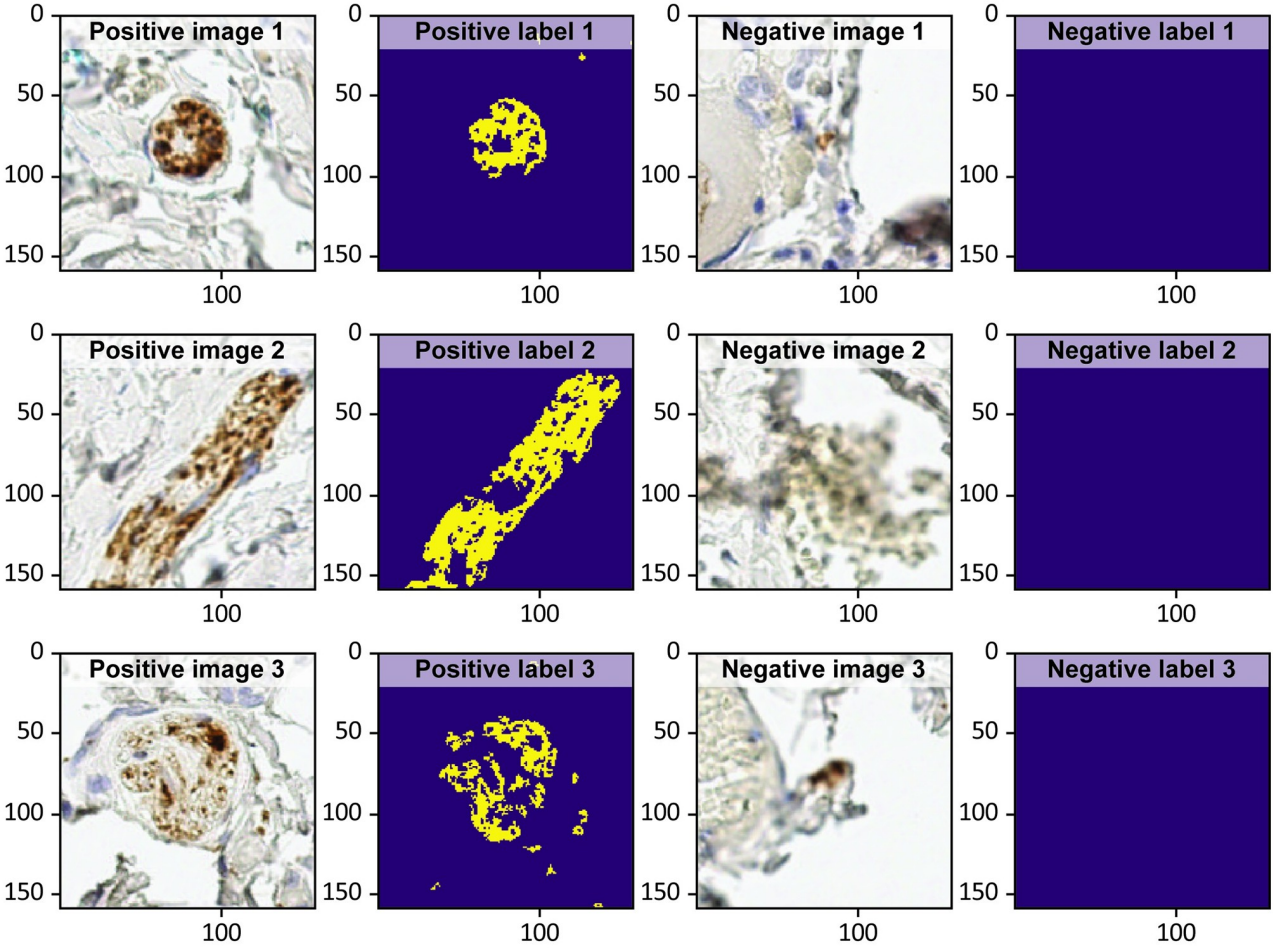
Examples of the positive and negative training data samples.

The He initialisation is used for the weight initialisation. The Adam optimiser is utilised in the model training using a learning rate of 10^−4^ with a binary cross-entropy cost function. The model is trained over 250 epochs with the ∼2000 handpicked positive training data samples. A few examples of both the positive and negative training data samples and their corresponding labels are shown in Fig 12.

## 4 Results

In this section, we explain how the dataset is used and divided for the development of the model, as well as the test results at a WSI level. The results of the proposed approach are compared with the results from the manual counting and traditional automatic approach, i.e. colour filter based approach.

### 4.1 Dataset

The dataset consists of 112 stained thyroid tissue WSIs annotated by two experts. The dataset is split into training, validation and test sets. The training set consists of 80 WSIs, which includes 52 WSIs containing the largest number of annotated nerves and 28 randomly selected WSIs. The validation set consists of 10 randomly selected WSIs, while the test set consists of the remaining 22 WSIs.

### 4.2 Performance comparison

In this section, we present a performance comparison between manual counting by experts and two automatic approaches, i.e. the colour filter and the proposed CNN based approaches. The colour filter based approach (APR-CF) relies on a colour filter, while the CNN based approach (APR-CNN) relies on a CNN to perform a pixel-level nerve detection, as described in Section 3.6. Here, the CNN based approach uses the proposed U-Net with the augmented classification structure, as shown in Fig 6. The main objective of this section is to evaluate the proposed CNN based approach in terms of performance for automatic nerve detection.

Sensitivity (*TPR*) and precision (*PPV*) are used for performance evaluation of the three approaches on each WSI in the test set. The evaluation scores of the corresponding metrics for each WSI are presented in Tables 3 and 4. Due to the large number of predicted positive instances by the APR-CF, we use a random sampling to obtain 100 predictions for each WSI to facilitate the experts in the evaluation of the true positives. The performance evaluation of the APR-CF in Table 4 will be based on these 100 samples, where the proportion of correct predictions will be multiplied by the total number of predictions, and hence denoted as estimated performance.

**Table 3:** Results with respect to the expert manual annotations.

WSI ID	Manual Annotations	Identified manual annotations				Additional detections	
		APR-CF		APR-CNN			
		*TP*_*m*_	*TPR*	*TP*_*m*_	*TPR*	APR-CF	APR-CNN
**10012**	10	9	0.9	9	0.9	847	50
**10023**	12	12	1	11	0.92	2658	63
**10029**	11	11	1	8	0.73	3390	21
**10036**	9	8	0.89	9	1	4316	385
**10039**	35	35	1	31	0.89	6591	419
**10049**	15	15	1	12	0.8	7185	26
**10064**	28	28	1	25	0.89	1922	33
**10067**	27	27	1	25	0.93	815	146
**10071**	21	20	0.95	18	0.86	3113	40
**10072**	15	14	0.93	14	0.93	492	45
**10073**	2	2	1	1	0.5	8348	25
**10078**	15	15	1	13	0.87	9884	99
**10087**	0	0	-	0	-	877	11
**10088**	8	8	1	8	1	1689	100
**10093**	11	11	1	11	1	2773	17
**10096**	1	1	1	1	1	1019	32
**10097**	19	18	0.95	18	0.95	6907	511
**10102**	25	25	1	24	0.96	4799	405
**10113**	16	15	0.94	14	0.88	1504	245
**10114**	14	14	1	13	0.93	6529	1059
**10116**	16	16	1	15	0.94	5005	318
**10121**	8	8	1	7	0.88	4278	296
**Overall**	**318**	**312**	**0.98**	**287**	**0.89**	**84941**	**4346**

**Abbreviations**: WSI: Whole Slide Image; APR-CF: Colour filter; APR-CNN: CNN based approach; *TP*_*m*_: True positives detected from the manual annotations; *TPR*: Sensitivity.

**Table 4:** Precision scores of the automatic approaches.

WSI ID	Additional detections						Total			
	APR-CF			APR-CNN			APR-CF		APR-CNN	
	Total	Est. *TP*_*a*_	Est. *FP*	Total	*TP*_*a*_	*FP*	*TP*	*PPV*	*TP*	*PPV*
**10012**	847	45	802	50	34	16	54	0.06	43	0.73
**10023**	2658	89	2569	63	39	24	101	0.04	50	0.68
**10029**	3390	0	3390	21	11	10	11	0	19	0.66
**10036**	4316	432	3884	385	304	81	440	0.1	313	0.79
**10039**	6591	712	5879	419	316	103	747	0.11	347	0.77
**10049**	7185	48	7137	26	15	11	63	0.01	27	0.71
**10064**	1922	26	1896	33	21	12	54	0.03	46	0.79
**10067**	815	244	571	146	126	20	271	0.32	151	0.88
**10071**	3113	197	2916	40	29	11	217	0.07	47	0.81
**10072**	492	77	415	45	37	8	91	0.18	51	0.86
**10073**	8348	167	8181	25	17	8	169	0.02	18	0.69
**10078**	9884	659	9225	99	70	29	674	0.07	83	0.74
**10087**	877	9	868	11	10	1	9	0.01	10	0.91
**10088**	1689	146	1543	100	70	30	154	0.09	78	0.72
**10093**	2773	55	2718	17	11	6	66	0.02	22	0.79
**10096**	1019	27	992	32	18	14	28	0.03	19	0.58
**10097**	6907	1036	5871	511	395	116	1054	0.15	413	0.78
**10102**	4799	720	4079	405	285	120	745	0.15	309	0.72
**10113**	1504	140	1364	245	185	60	155	0.1	199	0.77
**10114**	6529	1372	5157	1059	806	253	1386	0.21	819	0.76
**10116**	5005	233	4772	318	222	96	249	0.05	237	0.71
**10121**	4278	399	3879	296	198	98	407	0.09	205	0.68
**Overall**	**84941**	**6833**	**78108**	**4346**	**3219**	**1127**	**7145**	**0.09**	**3506**	**0.75**

**Abbreviations**: WSI: Whole Slide Image; APR-CF: Colour filter; APR-CNN: CNN based approach; Est.: Estimated performance based on 100 samples; *TP*_*a*_: The number of true positives in the additional detections; *FP*: False Positives; *TP*: Total true positives; *PPV*: Precision.

Table 3 provides the number of detected nerves for the APR-CF and APR-CNN with respect to the expert manual annotations. The identified manual annotations columns indicate the number of expert annotations that are detected by each approach. The additional detection columns indicate nerves detected by the approaches that were missed by the experts. Note, that the additional detections may contain false positives.

As can be seen from Table 3, the APR-CF has a higher rate of annotated nerve detection, with an average sensitivity of 98%, while the CNN based approach has an average sensitivity of 89%. However, the APR-CF predicted a total of 84,941 additional positive detections, while the CNN based approach predicted a total of 4346, which is more than an order of magnitude less than the APR-CF. However, the quality of the overall performance can only be determined by the evaluation of the true positives of these predictions.

Table 4 details the additional detections from the colour filter and CNN based approach in terms of true and false positives. It provides the number of nerves (i.e. true positives, *TP*_*a*_) that were not detected by expert manual annotation, and the number of predicted positive instances that were not nerves (i.e. false positives, *FP*). It also shows the precision of the automatic approaches.

As can be seen from Table 4, the automatic approaches are capable of detecting a significantly higher number of true positives, at least eleven times more than the number of nerves manually detected by the experts shown in Table 3. However, compared to the APR-CF, the APR-CNN performs substantially better in correctly identifying nerves (precision). The APR-CF has an average precision of 9%, while APR-CNN has an average precision of 75%. Although, the APR-CF has the highest number of additional positive detections, the number of false positives is extremely large, hence the detection is not meaningful. This significant improvement in precision makes the detection substantially more meaningful.

**Fig 13:**
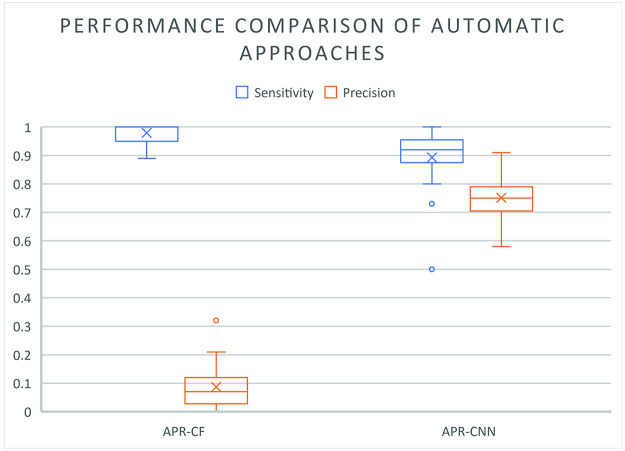
Sensitivity of the APR-CF and APR-CNN in the detection of the expert annotated nerves (blue). Precision of APR-CF and APR-CNN in the detection of nerves (orange). The × symbol in the box indicates the mean value, while the line in the box indicates the median value. The ∘ represents an outlier, the box represents the interquartile range and the whiskers represent the upper and lower extreme, excluding the outliers.

A summary of the sensitivity and precision of the automatic approaches, as evaluated on the test set, is presented in the box-and-whiskers plots shown in Fig 13 where it can be seen that APR-CNN outperforms the APR-CF.

**Fig 14:**
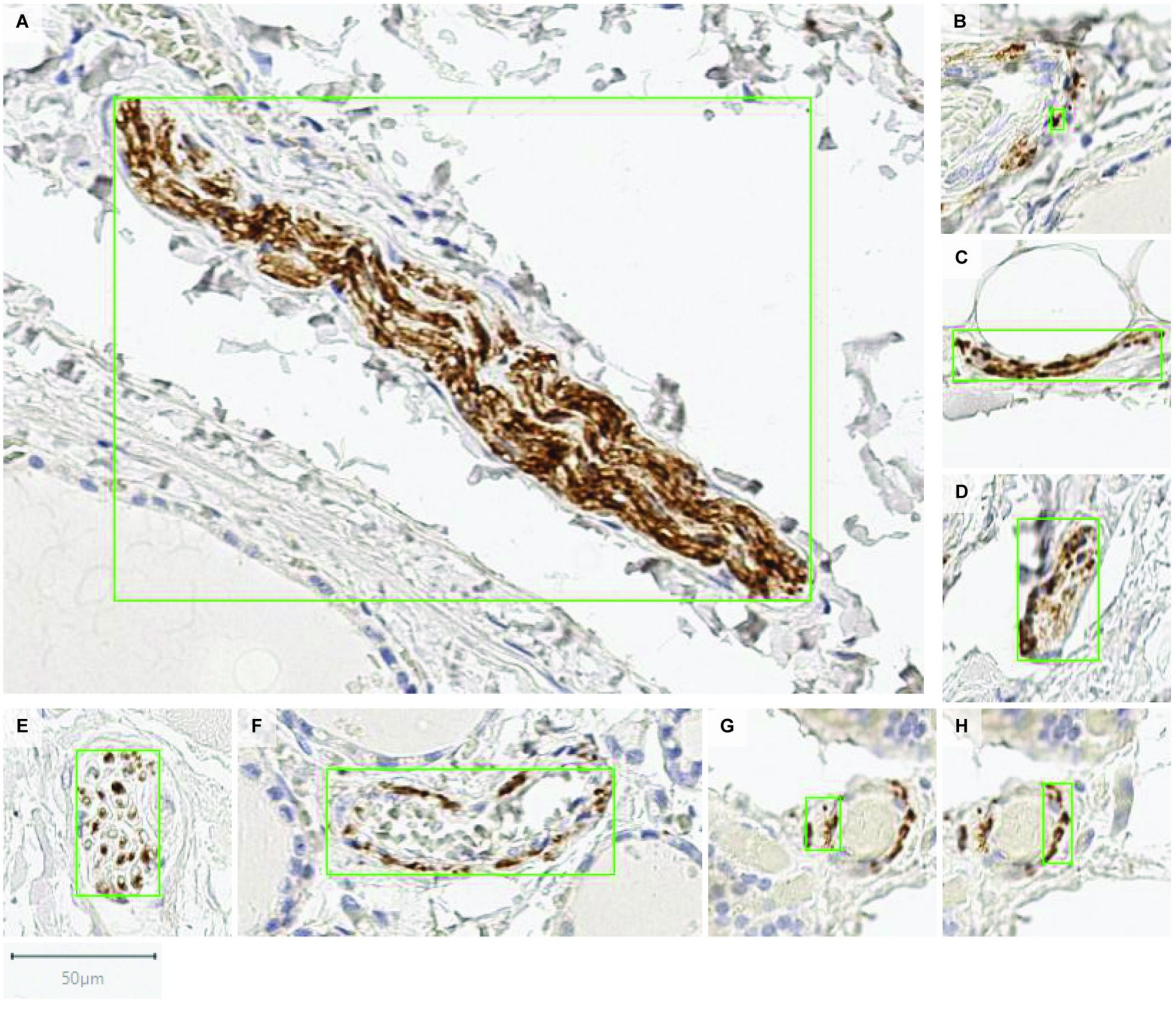
Examples of nerves detected (i.e. true positives, *TP*_*a*_) by the proposed CNN based approach. Each image patch contains one prediction instance indicated by a green box.

Several examples of true positives detected by the CNN approach are shown in Fig 14(A)–14(H). It can be seen that the CNN approach can detect nerves of various sizes and appearances. Here we present one of the largest nerves detected, 40.5 x 300 *μ*m^2^ (Fig 14A), and the smallest 4.5 x 7 *μ*m^2^ (Fig 14B). Nerves within the model input size (160 × 160 pixels) are shown in Fig 14(C)–14(E) and 14(G)–14(H), as well as larger nerves shown in Fig 14(A) and 14(F). A nerve in the form of a cluster of axons that are close to each other, considered as a single nerve, is shown in Fig 14(C)–14(F). Multiple clusters of axons that are far apart from each other are considered to be separate nerves and quantified as multiple discrete nerves, as shown in Fig 14(G)–14(H).

Overall, the experts detect the least number of nerves in the test set, with a high precision and low sensitivity. The APR-CF detects approximately 22 times the nerves detected by the experts, however, with an extremely low precision of 9% due to the large number of false positives. The CNN based approach detects approximately 11 times the number of nerves detected by the expert and has an average precision of 75%. This shows that the CNN based approach can detect considerably more nerves than the manual approach while having more meaningful results than the traditional colour filter based approach.

## 5 Discussion

In this paper, we propose an automated CNN based approach that is more suitable than either manual counting or a simple colour filter to perform nerve detection and quantification. We have shown that although the colour filter based approach is highly sensitive, it tends to have a very low precision. On the other hand, the proposed CNN based approach (APR-CNN) is slightly less sensitive than the colour filter based approach (APR-CF), but has a much greater precision which makes the detection considerably more meaningful.

Influence of nerves on tissues is a function of “nerve density”. More specifically the density of terminal fields and neurotransmitter release sites. An individual varicosity (release site) is around 1 micron in diameter. To catch every varicosity is unrealistic due to the fact that pathology specimens are sectioned 4 microns and the microtome blade will slice through parts of varicosities. So there has to be a compromise. As stated in the introduction manual counting has previously only been able to detect large nerve trunks. Essentially giving no information on nerve density within the tumour. The size filter employed in this study is conservative with respect to single varicosities and parts thereof. However, it is a vast improvement on any previous quantification of nerve density. As such it has the potential to significantly add to the understanding of cancer progression in these patients.

This proof of concept study, demonstrating machine learning techniques for detection of nerves in cancer tissues, is expected to be widely generalisable to detection of nerves in many cancer (and non-cancer) histology specimens. Thyroid cancer was chosen for this study as a pragmatic available dataset, however any appropriately prepared tissue-type could have been used.

Note that although the development was affected by the inconsistency of the experts due to intra- and inter-expert variability, we believe that the developed model will be able to minimise the intra- and inter-expert variability in future studies.

## 6 Conclusion

In this paper, we defined a detailed nerve quantification criteria and developed an automatic nerve detection system based on a CNN. We proposed a novel augmented classification structure for a U-Net to reduce the number of false positives in an object detection task. This new CNN based approach resulted in having a much higher precision score (75%) with respect to the traditional colour filter based approach (9%) while also being more consistent than the manual counting. The proposed approach resulted in an increase of the nerve detection capability of approximately 11 times with respect to manual counting by the experts, while maintaining an 89% sensitivity.
